# Analyzing the Challenges and Opportunities Associated With Harnessing New Antibiotics From the Fungal Microbiome

**DOI:** 10.1002/mbo3.70034

**Published:** 2025-07-23

**Authors:** Md. Sakhawat Hossain, Md. Al Amin, Sirajul Islam, Hasan Imam, Liton Chandra Das, Shahin Mahmud

**Affiliations:** ^1^ Department of Biotechnology and Genetic Engineering Mawlana Bhashani Science and Technology University Santosh Tangail Bangladesh; ^2^ Department of Biochemistry and Molecular Biology Siddheswari College Moghbazar Dhaka Bangladesh

**Keywords:** antibiotic discovery, fungal metabolite sharing, fungal microbiome, fungal–bacterial interactions, industrial‐scale processes of antibiotics

## Abstract

The rapid rise in antibiotic resistance is a critical global health issue, and few new classes of antibiotics have been discovered since 1990 compared to the antibiotic's golden era between 1950 and 1970. However, developing new antimicrobial compounds faces many challenges, improvements in cultivation methods, genetic engineering, and advanced technologies are opening new paths for discovering and producing effective antibiotics. This study focuses on the fungal microbiome as a promising source of new antibiotics. We explored historical developments and advanced genetic techniques to reveal the potential of fungi in antibiotic production. Although isolating and scaling up fungal antibiotic production presents challenges, innovative approaches like in situ separation during fermentation can effectively address these issues. Our research highlights the importance of understanding fungal communication and metabolite sharing to enhance antibiotic yields and the connection of cutting‐edge technologies in accelerating the discovery and optimization of antibiotic‐producing fungi. By focusing on these technical aspects and fostering teamwork across various fields, this study aims to overcome current obstacles, and advance the development of antibiotic production technologies.

## Graphical abstract

Graphical abstract illustrating the multifaceted approach to harnessing fungal microbiome diversity for antibiotic discovery. Key elements include historical insights, modern genomic tools, and innovative biotechnological strategies for isolating and enhancing fungal antibiotic production. Highlighted are fungal communication networks and advanced fermentation techniques aimed at scaling up production and improving antibiotic yields.

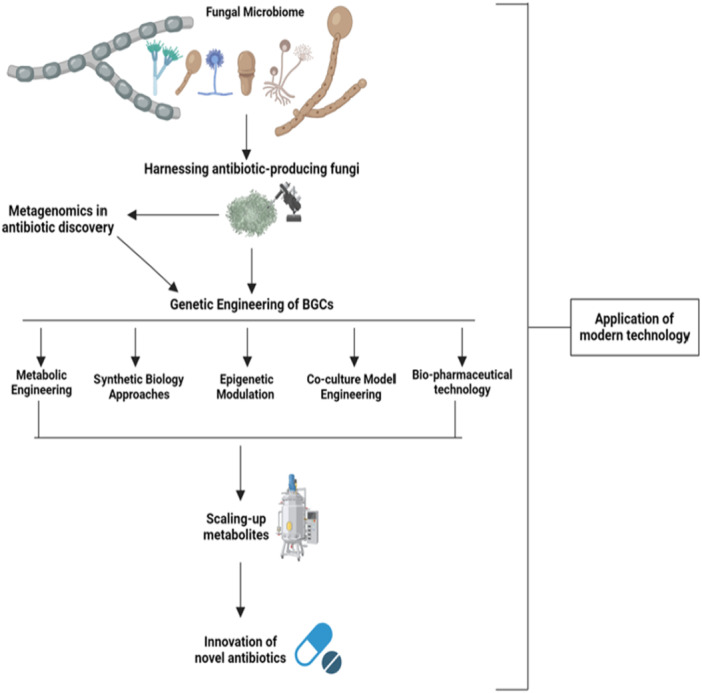

## 1 Introduction

The global rise of antimicrobial resistance has created an urgent demand for new antibiotics, yet the pace of discovering novel compounds has significantly slowed in recent decades (Salam et al. 2023). One often‐overlooked but immensely promising source is the fungal microbiome, which continues to reveal its potential as a reservoir of bioactive secondary metabolites with potent antimicrobial properties (A. Gupta et al. 2023). Fungi have long been recognized for their vital role in antibiotic discovery and ation to the development of essential drugs that have revolutionized modern medicine (Walker 2021; Dutta et al. 2022). From the serendipitous discovery of penicillin to the synthesis of blockbuster drugs like Cyclosporine and Caspofungin, fungi have provided a rich reservoir of secondary metabolites with potent bioactive properties (Baby and Thomas 2021). The unique biosynthetic capabilities of fungi, often stimulated by environmental stress factors, enable them to produce diverse bioactive compounds (Saxena et al. 2018). Despite this rich legacy, the pace of discovering new antibiotics has slowed, prompting a reevaluation of traditional bioprospecting methods (Ryan et al. 2019). This has led to a shift in focus toward understanding the ecological and genetic underpinnings of fungal metabolite production and leveraging cutting‐edge tools to unlock previously inaccessible biosynthetic capacities (Alves et al. 2025).

In recent years, advances in genomic and metabolomic technologies have revitalized interest in fungal secondary metabolites (Jampilek 2022). Techniques such as whole‐genome sequencing and bioinformatics tools have enabled the identification of novel biosynthetic gene clusters (BGCs), facilitating the dereplication process and reducing rediscovery rates (Karwehl and Stadler 2016). Additionally, advances in chromatographic, spectroscopic, and fermentation techniques have facilitated the isolation and characterization of countless new metabolites (Silber et al. 2016). Coculture techniques and stress‐induced biosynthetic pathways have emerged as innovative strategies to uncover hidden bioactive compounds, further expanding the scope of fungal antibiotic discovery (Demers et al. 2018; Krause 2021). The urgency to address antimicrobial resistance has underscored the need for new therapeutic agents, driving researchers to explore untapped fungal biodiversity using modern “‐OMICS” technologies and advanced biotechnological methods (Snelders et al. 2020). Techniques like CRISPR‐Cas9 genome editing, promoter engineering, and heterologous expression systems are helping researchers tap into the hidden chemical diversity of fungi (Leal et al. 2024). Studies have shown that fungi produce specialized metabolites with potent antimicrobial properties that influence bacterial community diversity in natural environments (Mosunova et al. 2022). The human microbiome itself, including fungal components, has been shown to harbor diverse microbial communities that produce bioactive molecules with potential therapeutic applications (Xiong 2016). As researchers continue to explore extreme habitats and symbiotic associations, the integration of interdisciplinary approaches and advanced technologies holds promise for harnessing the fungal microbiome as a rich reservoir of novel antibiotics (Nirmala and Zyju 2017). Beyond laboratory innovations, understanding fungal communication with bacteria and exploring fungal–bacterial co‐cultures have opened exciting possibilities for boosting metabolite production and discovering novel compounds (Hyde et al. 2024). These interdisciplinary efforts are reshaping how we approach fungal microbiome research for antibiotic discovery (Tiew et al. 2020).

In this review article, we investigated various aspects of fungal microbiome research aimed at uncovering novel antimicrobial compounds. We explored historical developments in fungal antibiotic discovery to challenges associated with isolating BGCs from fungal genomes and discussed strategies for identifying antibiotic‐producing fungi from complex microbial communities.

## 2 Fungal Microbiome in Antibiotic Discovery

Research into fungal microbiomes has greatly advanced antibiotic development and uncovered many potential bioactive compounds. Filamentous fungi, such as those from the *Penicillium* genus, are prolific producers of secondary metabolites with diverse pharmaceutical applications, including antibiotics, immunosuppressors, and anticancer drugs (Dutta et al. 2022; de Matos et al. 2023). The discovery of penicillin marked the beginning of the golden era of antibiotics, and fungi have since provided numerous blockbuster drugs like Cyclosporine and Lovastatin (Prescott et al. 2023). Marine fungi, in particular, have demonstrated vast chemo diversity and potential for sustainable antibiotic production through marine biotechnology (Suresh and Abinayalakshmi 2022). Advances in genomics and metabolomics have further accelerated the discovery of novel antibiotics, revealing that the biosynthetic potential of fungi is far from exhausted (Panter et al. 2021). The manipulation of host microbiomes by fungal pathogens through effector proteins also presents an untapped resource for new antibiotics (Snelders et al. 2020). The rich biodiversity and unique biosynthetic capabilities of fungi make them invaluable in the ongoing quest for new antibiotics to combat the rising threat of antimicrobial resistance (da Silva et al. 2023).

### 2.1 Historical Perspective: Fungal Contributions to Antibiotic Discovery

The discovery and development of antibiotics from the fungal microbiome have marked significant milestones in medical history. The discovery of penicillin from the mold *Penicillium notatum* by Alexander Fleming in 1928 marked the beginning of the antibiotic era (Fleming 1929). This landmark discovery spurred the exploration of fungal sources for novel antibiotics, leading to the identification of cephalosporins from *Cephalosporium acremonium* (Newton and Abraham 1955) in 1956 and griseofulvin from *Penicillium griseofulvum* in 1959 (Flint et al. 1959). These compounds have been crucial in treating various infections and have paved the way for modern antimicrobial therapy.

Recent advancements in genomics and bioinformatics have reignited interest in the fungal microbiome as a reservoir of novel bioactive compounds (Conrado et al. 2022). Techniques like metagenomics and genome mining have opened doors to previously inaccessible BGCs, facilitating the discovery of promising new antibiotic candidates (Clevenger et al. 2017). Several clinically relevant antibiotics currently in use originate from fungal sources. Table 1 provides a summary of these major antibiotics, including their source organism and their biological target. This ongoing research highlights the immense potential of fungal microbiomes to contribute to the development of the next generation of antibiotics.

**Table 1:** Clinically relevant antibiotics family isolated directly from fungal microbiome.

Antibiotic class	Example of clinically used drug	Biological target	Fungus name	Reference
Penicillins	Penicillin G	Bacterial cell wall synthesis	*Penicillium notatum*	Broomfield and Filipa Simões (2019)
Cephalosporins	Cefadroxil	Bacterial cell wall synthesis	*Acremonium chrysogenum* (formerly *Cephalosporium acremonium*)	L. Liu, Chen et al. (2022)
Pleuromutilins	Retapamulin	Protein synthesis	*Clitopilus passeckerianus*	de Mattos‐Shipley et al. (2017)
Amoxicillin	Clavacillin	Protein synthesis	*Aspergillus clavatus*	Katzman et al. (1944)
Carbapenems	Imipenem	Bacterial cell wall synthesis	*Streptomyces cattleya* (fungal‐associated)	Imipenem (2025)
Glycopeptides	Vancomycin	Bacterial cell wall synthesis	*Mycolatopsis orientalis*	Amycolatopsis Orientalis (2025)
Macrolides	Erythromycin	Protein synthesis (50S ribosomal subunit)	*Saccharopolyspora erythraea*	Dinos (2017)
Ansamycins	Rifampin	RNA polymerase inhibition	*Amycolatopsis rifamycinica*	Ansamycin (2025)
Tetracyclines	Doxycycline	Protein synthesis (30S ribosomal subunit)	*Streptomyces* spp.	Tetracycline (2025)

### 2.2 Fungal Microbiome: A Reservoir of Bioactive Potential

The fungal microbiome is indeed a significant reservoir of bioactive potential, offering a plethora of compounds with diverse applications in pharmaceuticals, agriculture, and biotechnology. Fungi, including endophytes, basidiomycetes, and actinomycetes, produce a wide range of bioactive metabolites such as steroids, terpenoids, quinones, coumarins, phenols, saponins, and alkaloids, which exhibit anticancer, immunomodulatory, antitubercular, antiviral, and antidiabetic activities (Pant et al. 2021; Rousta et al. 2023). These metabolites are crucial for developing new drugs and therapies, addressing the high demand for novel antimicrobial and antitumor agents (Singh 2021). Fungal strains are also known for synthesizing bioactive compounds like bioactive peptides, chitin/chitosan, β‐glucan, ɣ‐aminobutyric acid, l‐carnitine, ergosterol, and fructooligosaccharides, which have significant health benefits and potential applications in innovative food production (Chourasia et al. 2022; Shahbaz et al. 2023). The exploration of fungal endophytes, which live symbiotically within plant tissues, has revealed their potential to produce unique bioactive compounds that can improve plant health and offer therapeutic benefits to humans (Selvakumar and Panneerselvam 2018).

### 2.3 The Potentiality of Fungal Secondary Metabolites

Fungi exhibit high productivity in the synthesis of bioactive secondary metabolites, which hold considerable promise for advancement in the field of antimicrobial drug discovery (Table 2). For instance, *Nigrospora* spp. isolated from *Rhizophora racemosa* produces a variety of compounds with antimicrobial properties against both Gram‐positive and Gram‐negative bacteria (Chourasia et al. 2022). *Aspergillus polyporicola*, isolated from *Synsepalum dulcificum* roots, yields several new and known compounds that exhibit inhibitory activities against Methicillin‐resistant *Staphylococcus aureus* (MRSA), *Staphylococcus aureus*, and other pathogens (S. S. Liu, Huang et al. 2022). Similarly, secondary metabolites from *Aspergillus niger* and *Trichoderma lixii* show high antimicrobial activity, particularly against *Staphylococcus aureus* and *E. coli*, and demonstrate a synergistic effect when combined with dicationic pyridinium iodide against *Klebsiella pneumoniae* (Abdelalatif et al. 2023). *Chaetomium elatum*, isolated from *Hyssopus officinalis*, produces penicillic acid, which has strong antibacterial effects against *Bacillus subtilis* and *Staphylococcus aureus* (Soytong and Kanokmedhakul 2022). Additionally, *Aspergillus sydowii* from seawater yields compounds with selective inhibitory activities against several human pathogenic bacteria (Gao et al. 2017).

**Table 2:** List of high‐productivity bioactive secondary metabolites isolated from fungus for antimicrobial drug discovery.

Fungal source	Type of fungi	Secondary metabolites Identified	Antimicrobial activities	Reference
*Nigrospora* spp.	Endophytic	Septicine, aureonitol, papuamine, di‐iso‐octylphthalate, cladosporin, tetrabenzofuran, eicosane.	Antimicrobial activities against Gram‐positive and Gram‐negative bacteria.	Izuogu et al. (2023)
*Aspergillus polyporicola*	Saprophytic	Kipukasins O and P, arthropsadiol D, (+)‐2,5‐dimethyl‐3(2H)‐benzofuranone, polyporicolic acids A and B, (+)‐acetylkojic acid, and others.	Inhibitory activity against MRSA, *Staphylococcus aureus*, Salmonella typhimurium, and fungi.	S. Liu, Huang et al. (2022)
*Aspergillus niger* and *Trichoderma lixii*	Saprophytic	Pentadecanoic acid, 14‐methyl‐, methyl ester, 9‐octadecenoic acid (Z)‐, methyl ester, 2,4‐decadienal, 1,8‐cineole, 4‐hydroxybenzaldehyde, and others.	High activity against *Staphylococcus aureus* and *E. coli*; synergistic effect with dicationic pyridinium iodide against *Klebsiella pneumoniae*.	Abdelalatif et al. (2023)
*Chaetomium elatum*	Endophytic	Penicillic acid, Chaetoglobosin A, Chaetoglobosin C.	Strong antibacterial activities against Bacillus subtilis and *Staphylococcus aureus*.	Eshboev et al. (2023)
*Aspergillus sydowii*	Saprophytic/endophytic	Quinazolinone alkaloid, aromatic bisabolene‐type sesquiterpenoid, chorismic acid analogue, sydowic acid, sydonic acid.	Selective inhibitory activities against *Escherichia coli*, *Staphylococcus aureus*, *S. epidermidis*, and *Streptococcus pneumoniae*.	Gao et al. (2017)
*Colletotrichum* sp.	Saprophytic/endophytic	Acropyrone, beauvericin, indole‐3‐carbaldehyde, indolyl‐3‐acetic acid, rocaglamid A.	Inhibitory effect against Bacillus subtilis, Pseudomonas aeruginosa, Aspergillus niger, *Staphylococcus aureus*, *E. coli*.	Munasinghe et al. (2017)
*Candida* spp.	Commensal	Benzene, pentamethyl, n‐hexadecanoic acid, 1‐docosene, bis(2‐ethylhexyl), others.	Various antimicrobial activities.	Hassan and Kasim (2023)

### 2.4 Advances in Fungal Metabolic and Genetic Engineering for Antibiotic Discovery

**Figure 1:**
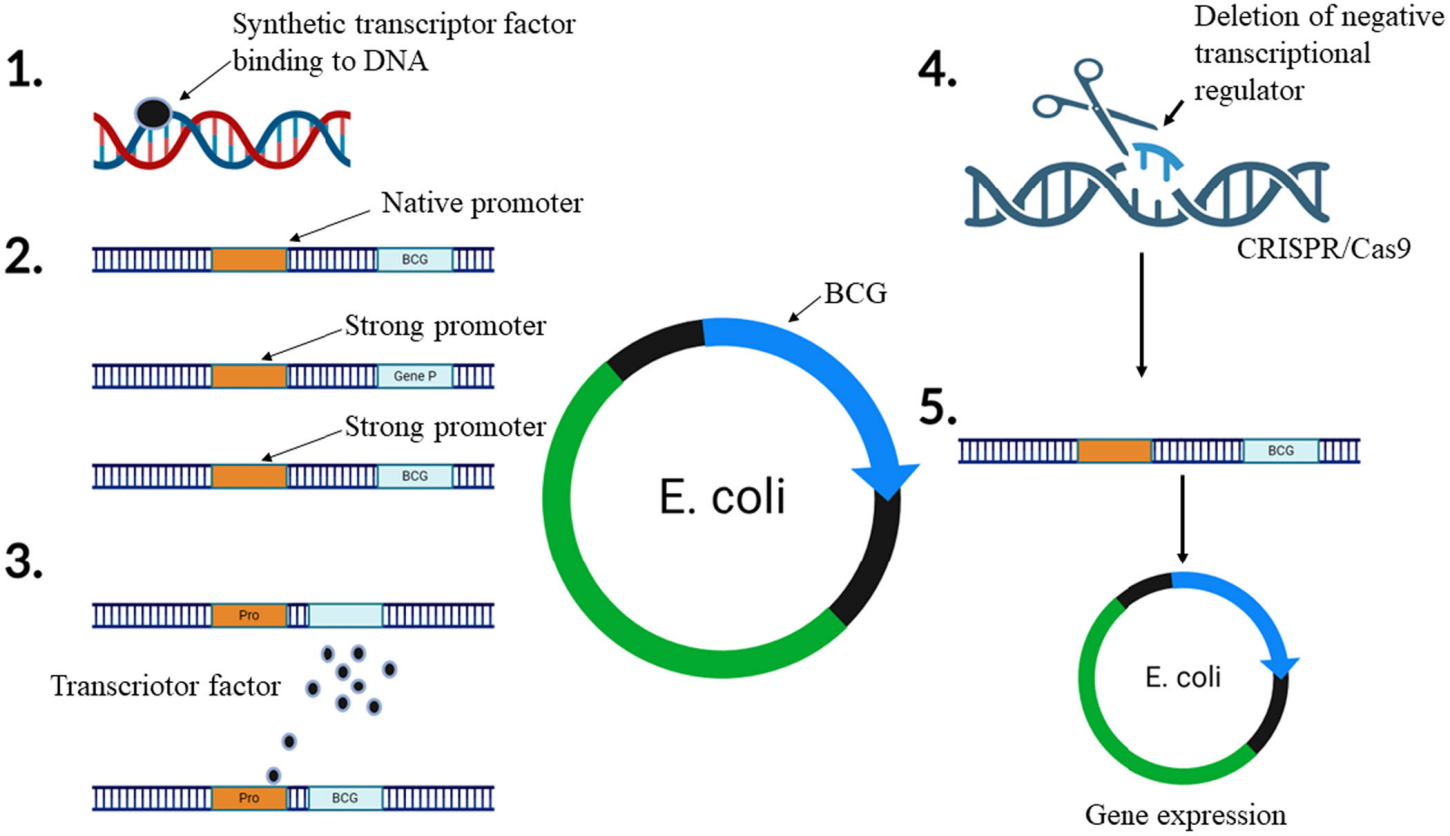
Potential approaches for enhancing the heterologous expression of biosynthetic gene clusters (BGCs) in Fungi.

Metabolic engineering can significantly enhance fungal biosynthesis by manipulating and optimizing various biosynthetic pathways to increase the yield and efficiency of desired metabolites. Advances in DNA sequencing and bioinformatics have revealed numerous cryptic BGCs in fungal genomes, which are often transcriptionally silent under laboratory conditions (Hur et al. 2023; D. Wang, Jin, et al. 2023). Strategies to “waking up” silent gene clusters for antibiotic production include the use of synthetic transcription factors, gene cluster refactoring, and heterologous expression in optimized host strains strains such as *Aspergillus oryzae* and *Fusarium graminearum*. The different methods are outlined in Figure 1.

#### 2.4.1 Synthetic Biology Approaches

Synthetic biology tools, including synthetic transcription factors, gene cluster refactoring, and the assembly of artificial transcription units, further enhance the expression of both endogenous and exogenous BGCs in filamentous fungi (Mózsik, Pohl, et al. 2021). Targeted overexpression of pathway‐specific transcriptional regulators, such as AspE in *Aspergillus* sp., has resulted in the activation of previously silent metabolic pathways and the production of novel bioactive compounds with significant antimicrobial properties (Etxebeste 2021). Epigenetic modifications using small molecular compounds to alter chromatin structure have also shown promise in activating silent BGCs, although the response can be complex and variable (Pillay et al. 2022). Furthermore, coexpression network approaches have been utilized to identify and overexpress global and pathway‐specific transcription factors, thereby modulating secondary metabolite production in fungi like *Aspergillus niger* (Cairns et al. 2019).

#### 2.4.2 Promoter Engineering

Promoter exchange techniques have become an effective method to activate BGCs in antibiotic‐producing fungi by replacing native promoters with strong, inducible ones to overcome the issue of silent or low‐expressing gene clusters in standard lab conditions, while an “always‐on” promoter ensures constant expression of antibiotic biosynthetic genes (Bergmann et al. 2010). For instance, the easyPACId method demonstrated that exchanging native promoters with inducible ones in Δhfq mutants of *Photorhabdus*, *Xenorhabdus*, and *Pseudomonas* led to the exclusive production of corresponding natural products (NPs) from targeted BGCs, facilitating NP identification and bioactivity testing (Bode et al. 2019). Additionally, the use of endogenous promoter libraries, constructed through rational design and site‐selective mutagenesis, has been shown to optimize multi‐gene metabolic pathways for high‐yield production of metabolites (Jin et al. 2019). Replacing a native promoter with the highly inducible alcA promoter in *Aspergillus nidulans* can boost penicillin production by up to 100 times, resulting in a significant 30‐fold increase in penicillin yields (Pi et al. 2020). Additionally, the use of constitutive promoters like gpdA and tef1 has been shown to enhance secondary metabolite production, indicating the potential of strong promoters in industrial applications (Umemura et al. 2020).

#### 2.4.3 Transcription Factor Overexpression

Overexpression of transcription factors represents a potent approach for the activation of BGCs within the fungal microbiome, consequently facilitating the synthesis of essential secondary metabolites. By overexpressing pathway‐specific transcription factors, researchers have successfully activated these silent clusters, leading to the discovery of novel compounds. For instance, overexpression of the transcriptional regulator AspE in *Aspergillus* sp. CPCC 400735 activated a downregulated metabolic pathway, resulting in the production of 13 asperphenalenone derivatives with significant anti‐influenza A virus effects (Khan et al. 2020). Similarly, the overexpression of the xan BGC transcription factor AfXanC in *Aspergillus fumigatus* significantly increased xanthocillin production, although its ortholog PeXanC in *Penicillium expansum* unexpectedly promoted citrinin synthesis instead (Paul et al. 2022). Additionally, global regulatory networks have been explored using coexpression network approaches, identifying multiple transcription factors that modulate secondary metabolism in *Aspergillus niger* (Kwon et al. 2021). Finally, the transcription factor TRI6 in *Fusarium graminearum* was shown to regulate multiple BGCs, both directly and indirectly, highlighting the complex regulatory networks governing secondary metabolism (Shostak et al. 2020).

#### 2.4.4 CRISPR/Cas9 Genome Editing

The CRISPR/Cas9 system, characterized by its ability to introduce precise genetic modifications, has been effectively employed to activate silent BGCs. The versatility of CRISPR/Cas9 extends to various Cas proteins, such as Cas12, which have been explored for their unique properties in genome editing across different fungal species (Agarwal 2023). The deletion of the negative transcriptional regulator mcrA in *Aspergillus wentii* led to the differential production of various secondary metabolites. This process revealed new compounds through additional genetic modifications (Yuan et al. 2022). Additionally, the system has been applied to confirm the involvement of specific gene clusters in secondary metabolites biosynthesis, as demonstrated in *Lophiotrema* sp. F6932, where targeted deletion of the ketosynthase domain in the PAL gene cluster halted the production of palmarumycins (Kwon et al. 2021). Moreover, Innovative approaches like CRISPR activation (CRISPRa) using dCas9‐VPR have further streamlined the activation of silent gene clusters, exemplified by the production of antimicrobial macrophorins in *Penicillium rubens* (Mózsik, Hoekzema, et al. 2021). The CRISPR/Cas12a‐mediated system, known as CAT‐FISHING, has been developed to efficiently clone large BGCs, facilitating the discovery of new bioactive compounds (Liang et al. 2022). Furthermore, the use of Cas9‐based RNA‐guided synthetic transcription activation systems, such as VPR‐dCas9, has proven effective in activating silent BGCs in *Aspergillus nidulans*, even in regions of facultative heterochromatin (Nan et al. 2021; Schüller et al. 2020). This approach is helping scientists to explore bigger and more complex clusters that were previously too difficult to work with. In addition to activating new pathways, CRISPR tools are also being used to improve production yields (Ansori et al. 2023). For example, by knocking down competing metabolic pathways using CRISPR interference (CRISPRi), fungi can be guided to focus more resources on producing the desired antimicrobial compounds (Zhao et al. 2021). Multiplexed CRISPR strategies, where several genes are edited or activated simultaneously, are also making it easier to reconstruct entire biosynthetic pathways in a more streamlined way (Zhao et al. 2021).

Overall, the CRISPR/Cas systems are reshaping how we approach fungal secondary metabolism. From activating silent clusters to optimizing production, these tools are creating exciting opportunities to discover new antibiotics and bioactive compounds from fungal sources.

#### 2.4.5 Heterologous Expression

Heterologous expression approach involves transferring BGCs from their native, often genetically intractable or difficult‐to‐cultivate hosts, into more manageable organisms like *Saccharomyces cerevisiae, Aspergillus nidulans*, or *Pichia pastoris* (Geistodt‐Kiener et al. 2023; Mózsik et al. 2022; Qian et al. 2022). Techniques such as transformation‐assisted recombination and polycistronic vectors have been developed to facilitate the seamless cloning and expression of these gene clusters in heterologous hosts (Y. Xu et al. 2022). For instance, the use of AMA1‐based pYFAC vectors in *Aspergillus nidulans* has shown high transformation efficiency and compound production, allowing for the evaluation of different pathway intermediates (Roux and Chooi 2022; Kanematsu and Shimizu 2015). Additionally, the refactoring of biosynthetic pathways in engineered hosts like *Aspergillus nidulans* and *Aspergillus oryzae* has led to the successful production of new compounds and the elucidation of biosynthetic pathways (Roux and Chooi 2022; Han et al. 2023). In Figure 1, we visualized the possible modern techniques to increase the expression of BGCs in fungi and boost the production of antibiotics through genetic methods.

### 2.5 Challenges in Harnessing BGCs From Fungal Genomics

Harnessing BGCs from fungal genomics presents several challenges despite the vast potential for discovering novel bioactive compounds. One primary issue is the difficulty in translating computational predictions of BGCs into actual compounds, as many BGCs remain transcriptionally silent under laboratory conditions, making it hard to identify and activate these pathways (Valiante 2023; D. Wang, Jin, et al. 2023). The regulatory circuits governing the expression of these gene clusters are often unknown, complicating efforts to exploit them in the lab (Keller 2019). Additionally, the sheer volume of data from over 1000 sequenced fungal genomes has yet to be systematically analyzed, which hampers the rational discovery of new compounds (Mohanta and Al‐Harrasi 2021; Schüller et al. 2022). Strategies like synthetic biology approaches are still in their infancy and require further refinement. Co‐expression network approaches need to be integrated more broadly into drug discovery programs to move beyond the BGC paradigm and understand the global regulatory networks involved (Soberanes‐Gutiérrez et al. 2022; S. Xu et al. 2023). Moreover, current BGC discovery tools often overestimate cluster boundaries, necessitating improved methods like reinforcement learning to optimize candidate BGC predictions (Yang et al. 2021; Almeida et al. 2022). While significant progress has been made, the challenges of activating silent BGCs, understanding their regulatory mechanisms, and efficiently mining the vast genomic data must be addressed to fully harness the biosynthetic potential of fungi.

### 2.6 Isolation and Identification of Antibiotic‐Producing Fungi

The isolation and identification of antibiotic‐producing fungi involve several environmental sources and steps, including sample collection, culturing, and screening for antimicrobial activity. Soil is a rich source of antibiotic‐producing microorganisms, including fungi, which can be isolated using various techniques such as serial dilution and spread plate methods on specific media like Sabouraud Dextrose Agar and Nutrient Agar (Broomfield and Filipa Simões 2019). For instance, fungi isolated from soil samples at Ahmadu Bello University included species like *Aspergillus niger, Aspergillus fumigatus*, and *Penicillium* sp., which showed antimicrobial activity against pathogens such as *Staphylococcus aureus* and *Escherichia coli* (Emmanuel and Igoche 2022).

**Figure 2:**
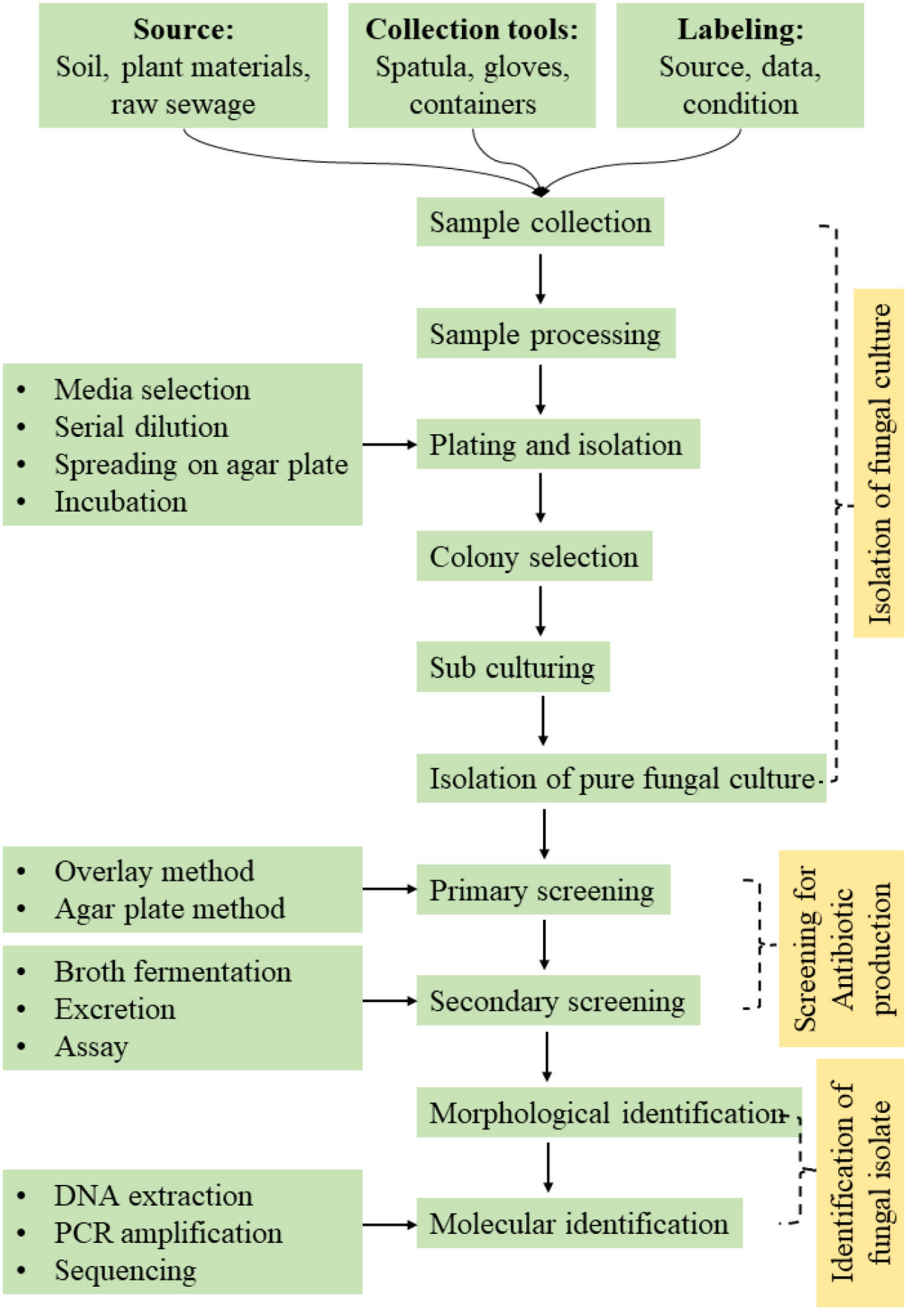
The generalized step‐by‐step process in isolating and discovering novel antibiotic‐producing fungi and characterizing their bioactive compounds.

Similarly, fungi from wastewater treatment plants and solid‐state waste sites have been found to produce antibiotics, with species like *Penicillium chrysogenum* and *Aspergillus flavus* demonstrating significant inhibitory effects against various bacteria (Verma and Haseena 2022; Dudeja et al. 2020). The identification of these fungi often involves both macroscopic and microscopic characterization, as well as molecular techniques like 18S rRNA gene sequencing (Logan et al. 2023). Additionally, fungi isolated from food products, such as *Aspergillus fumigatus* from stored sorghum grains, have been identified using molecular techniques (Prathibha et al. 2023). The antibiotic activity of isolated fungi is typically assessed using methods like the agar well diffusion assay and cross‐streaking techniques against a range of test microorganisms, including Gram‐positive and Gram‐negative bacteria (Singh et al. 2017; Yahaya et al. 2017). In Figure 2, we have developed a detailed process for identifying antibiotic‐producing fungi, which begins with sampling from various sources (Figure 2). These samples are then cultured under selective conditions designed to favor the growth of fungi with potential antibiotic activity.

The antibiotic activity of isolated fungi is initially assessed using classical methods like the agar well diffusion assay and cross‐streaking against Gram‐positive and Gram‐negative bacteria (Veeraswamy et al. 2022). However, to improve sensitivity, throughput, and reliability, advanced screening techniques are now being incorporated. Biosensors—analytical devices that combine biological recognition elements with signal transduction—enable real‐time, high‐sensitivity detection of bioactive metabolites (Tong et al. 2023). Meanwhile, high‐throughput screening allows rapid evaluation of large numbers of fungal extracts for antimicrobial properties using automated platforms (Ayon 2023). These modern tools significantly enhance the efficiency of discovering novel antibiotic‐producing strains.

### 2.7 Innovative Cultivation and Fermentation Strategies

Scaling up fungal production for antibiotic development involves transitioning from laboratory‐scale to industrial‐scale processes efficiently and cost‐effectively (Dutta et al. 2022). Successful scale‐up requires meticulous planning, attention to detail, and preparation for unforeseen challenges, especially during fermentation, which is a costly and critical step impacting downstream processing (Crater and Lievense 2018).

Cultivating fungi like *Aspergillus fumigatus* in various volumes to optimize secondary metabolite production is essential, with studies focusing on fermentation profiles and agitation speed effects (Tajabadi et al. 2015). Researchers have explored various techniques, the most efficient methods for the large‐scale cultivation of antibiotic‐producing fungi involve a combination of advanced cultivation techniques, optimized nutrient conditions, and innovative screening and monitoring technologies (Table 3). Another promising approach is aerobic submerged fermentation in a liquid medium with particulate anchoring carries has been shown to significantly increase mycelial biomass for large‐scale production (Giang et al. 2018; Farmer 2019). Similarly, microparticle‐enhanced cultivation (MPEC) techniques are particularly effective in overcoming the challenges of bulk fungal growth in bioreactors, improving homogeneity, and enhancing final product concentration without interfering with fungal metabolism (Mule et al. 2010). Different growth media compositions and physical conditions significantly influence the yield and potency of antibiotics produced by fungi. For instance, synthetic media with pure chemical sources are more consistent in penicillin production compared to semi‐synthetic media like potato dextrose agar (Waithaka 2022). The addition of biotic elicitors such as *Staphylococcus aureus* can extend the log phase growth of fungi like *Penicillium* sp., enhancing the antibiotic yield when added at specific times during fermentation (Fatima et al. 2019). Studies on *Micromonospora* sp. strain SMC23 revealed that specific solid media formulation could significantly enhance antibacterial metabolite production against ESKAPE pathogens (Giang et al. 2018).

**Table 3:** Scale‐up techniques for fungal antibiotic production.

Technique/strategy	Fungal strain involved	Reported enhancement in production	Reference
Aerobic submerged fermentation with anchoring carriers	*Aspergillus fumigatus*	2.5‐fold increase in biomass and metabolite yield	Yang et al. (2021), Almeida et al. (2022)
Microparticle‐enhanced cultivation (MPEC)	*Penicillium* sp.	1.8–2.2× increase in homogeneity and final product	Emmanuel and Igoche (2022)
Synthetic media (pure chemical) versus semi‐synthetic	*Penicillium chrysogenum*	~30% higher penicillin yield with synthetic media	Mubarak et al. (2022)
Biotic elicitor (*Staphylococcus aureus*) addition	*Penicillium* sp.	~1.5‐fold increase when added at optimal fermentation time	Verma and Haseena (2022)
Solid‐state fermentation with optimized media	*Micromonospora* sp. strain SMC23	~2.7× enhancement in activity against ESKAPE pathogens	Yang et al. (2021)
Agro‐waste media + glucose and NaNO_3_	*Aspergillus fumigatus*	~3.1× increase in antibiotic substance production	Dudeja et al. (2020)
Ultralow temp. strain storage (mutation prevention)	Multiple strains	Enhanced strain stability over long‐term runs	Logan et al. (2023)
In situ metabolite separation	Multiple fungal strains	Reduction in inhibitory metabolite accumulation	Prathibha et al. (2023), Singh et al. (2017)
CFD‐guided bioreactor design	Generic industrial fungi	Improved mass/heat transfer; scale‐up reproducibility	Ayon (2023), Crater and Lievense (2018)
PAT integration (Raman/MIR/UV–Vis)	*Aspergillus*, *Penicillium*	Improved real‐time control and fermentation yield	Waithaka (2022), Fatima et al. (2019), Chanphen et al. (2018)

**Figure 3:**
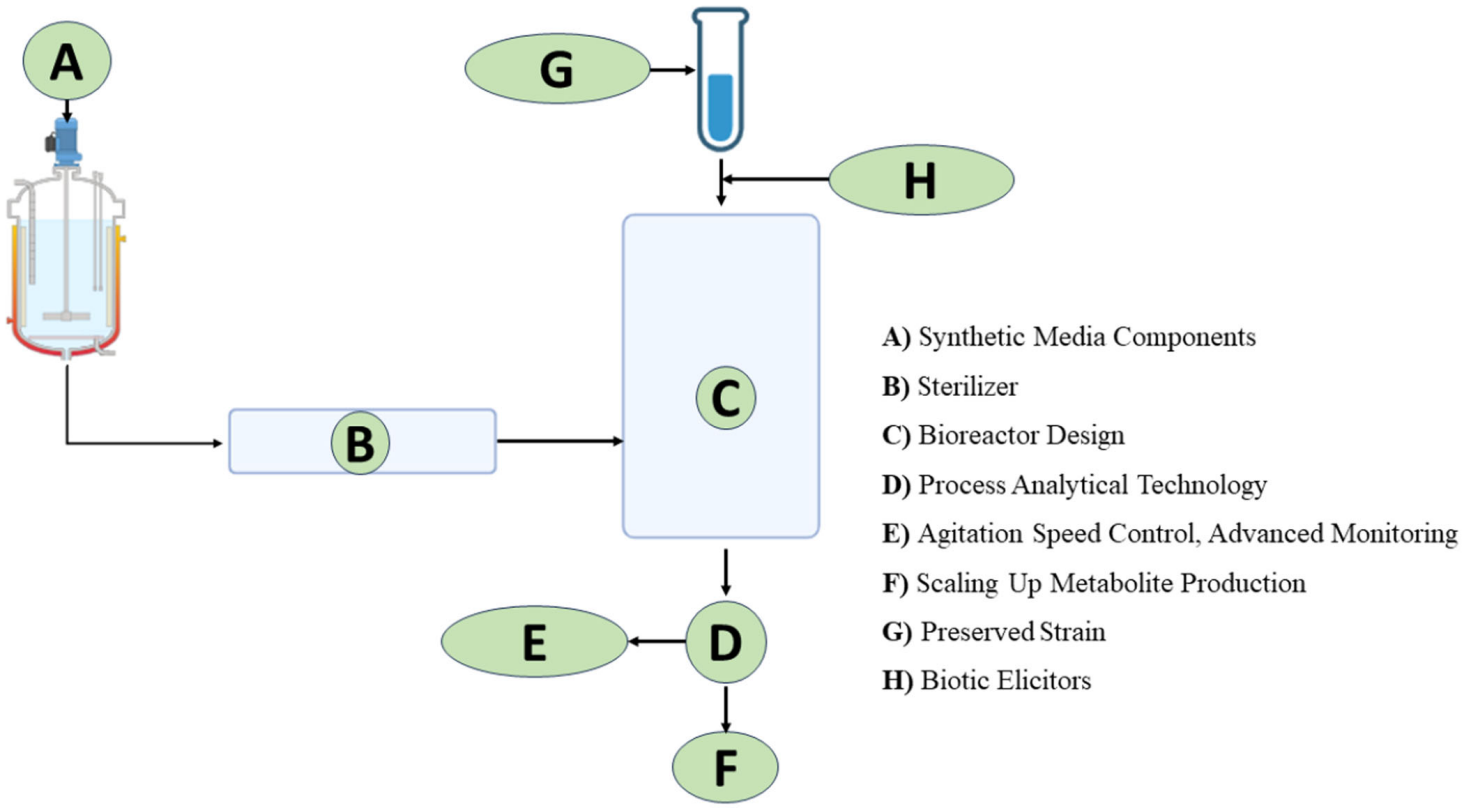
Procedures for scaling up fungal metabolite production in the biopharmaceutical industry.

Furthermore, exploring cost‐effective alternatives like the use of agro‐waste‐based media supplemented with glucose and NaNO_3_ at optimal pH and temperature conditions has been shown to support maximum antimicrobial substance production in fungi like *Aspergillus fumigatus* (Chanphen et al. 2018). Employing strain prevention might improve production quality, this method involves ultralow temperature storage, which helps maintain strain characteristics and prevent mutations (Serna‐Cock et al. 2012). Additionally, implementing in situ separations in the fermentation process can help remove inhibitory secondary metabolites that may affect DNA stability and lead to downregulated mutations (Tang and Ren 2011; Izzo et al. 2020). Effective bioreactor design for scaling up fungal antibiotic productions, which involves consideration of medium rheology, oxygen transfer, shear stress, and fungal morphology (Kırdök et al. 2022). Scale‐up processes are usually complex, requiring engineers to focus on factors like heat and mass transfer phenomena, medium composition, and mixing time (Gomes et al. 2022). Traditional designs such as stirred tank reactors and bubble columns are commonly used, but new configurations like solid‐state fermentation systems aim to address issues like shear stress and inefficient aeration (Mahdinia et al. 2019). Bioreactor scale‐up challenges are also related to transport processes, with scale‐down techniques and computational fluid dynamics simulations proving valuable in understanding heterogeneities observed in large‐scale tanks (Mayer et al. 2022; Liu 2017). Figure 3 represents the key steps involved in harnessing metabolites during the industrial process. This includes optimizing fungal cultivation conditions and implementing downstream processing for efficient metabolite extraction and purification.

Recent advancements focus on implementing control strategies tailored for industrial‐scale biotherapeutic production (Nikita et al. 2023), utilizing metabolic engineering techniques to increase antibiotic yield through genetic modifications and metabolic alterations (S. Gupta et al. 2020). However, the use of anaerobic membrane bioreactors shows promise in treating antibiotic wastewater effectively (Cheng et al. 2018), an optimization method based on Pontryagin's maximum principle and optimal control theory are also employed to determine the best control trajectories for key variables like temperature and substrate feed rate in antibiotic production bioprocesses (de Oliveira 2018).

Process analytical technology (PAT) can be integrated into fungal fermentation processes to enhance scalability and consistency by utilizing advanced monitoring strategies such as spectroscopic measurements and in situ approaches. By employing tools like mid‐infrared (MIR) spectroscopy (McDermott 2023) and in situ Raman and UV/Vis spectroscopy (Gerzon et al. 2022), real‐time monitoring of key parameters like glucose, ethanol, glycerol, and other analytes can be achieved, leading to improved process understanding, control, and reactor efficacy (Pontius et al. 2020). Updating and managing prediction models based on PAT data is crucial to account for variations in the process (Rößler et al. 2020). This integration of PAT not only aligns with modern paradigms like circular economy and sustainability but also supports the development of fungal fermentation processes (Aramouni et al. 2023).

Modern studies using tools like Design‐Expert software, Plackett‐Burman design, and response surface methodology have been employed to identify key factors influencing antibiotic production (Giang et al. 2018). Additionally, strategies like extractive fermentation via in situ ion‐exchange‐based absorptive techniques have been developed to mitigate feedback inhibition and enhance fermentation performance (Talukdar et al. 2016). Overall, these combined approaches may contribute to the efficacy and scalability of fungal antibiotic production processes on an industrial scale.

### 2.8 Fungal Communication and Regulatory Mechanisms in Antibiotic Development

#### 2.8.1 Quorum Sensing and Synthetic Inducers

Quorum Sensing (QS) is a cell‐density‐dependent signaling mechanism that fungi use to regulate various physiological processes, particularly for secondary metabolite production, including antibiotic biosynthesis (Padder et al. 2018). QS involves the release and detection of signaling molecules called autoinducers, such as farnesol and tyrosol (Rodrigues and Černáková 2020). When these molecules reach a critical concentration, they trigger changes in gene expression that activate key mechanisms like MAPK pathway proteins, cAMP–PKA pathways proteins, and BGCs, these pathways collectively boost antibiotic biosynthesis under certain conditions (Li et al. 2023; Polke et al. 2018; Santos‐Pascual et al. 2023).

To improve antibiotic yields, developing synthetic inducers (Kelly et al. 2016) that continuously activating these pathways under specific conditions could be a promising approach. Synthetic inducers are designed to mimic natural QS molecules but offer greater control over activation timing and intensity. For instance, replacing the promoter that derives the expression of transcriptional regulators in a common genome editing strategy to activate genes. This includes overexpressing activators, knocking out repressors, and modifying epigenetic modulators that control chromatin (Brakhage and Schroeckh 2011). While traditional genome editing can upregulate BGSs, recent advances in synthetic biology have introduced new tools like synthetic transcript factors, artificial transcription units, fungal shuttle vectors, and enhanced platform strains for heterologous expression (Mózsik et al. 2022).

#### 2.8.2 Epigenetic Regulation of Antibiotic Production

Epigenetic regulations significantly impact antibiotic production or BGC expression in fungi through mechanisms such as histone modification, DNA methylation, chromatin remodeling, and noncoding RNAs (Xue et al. 2023; Zhgun 2023; X. Wang, Yu, et al. 2023). Histone acetyltransferases (HATs) and histone deacetylases (HDACs) alter histone acetylation to regulate gene expression, with HDAC inhibitors like vorinostat and sodium butyrate enhancing antibiotic biosynthesis by opening chromatin structure (Giang et al. 2018; Fischer et al. 2017). Similarly, DNA methyltransferases add repressive methyl groups to DNA, while DNA demethylating agents like 5‐azacytidine can reactivate such silent BGCs and secondary metabolites production genes (Wu and Yu 2015). Additionally, chromatin remodeling complexes such as SWI/SNF adjust nucleosome positioning and noncoding RNAs further modulate these processes by directing chromatin‐modifying enzymes to specific loci (Kuwahara et al. 2023; Morse et al. 2023). To boost antibiotic production, focusing on novel epigenetic modulators—like HDAC and HMT inhibitors, DNA methylating agents, and synthetic epigenetic modulators—can provide precise control over fungal secondary metabolite gene expression. Synthetic epigenetic modulators represent a cutting‐edge research field that precisely controls gene expression through targeted modification of epigenetic marks (Carosso et al. 2023; Rittiner et al. 2022). However, delivering these modulators into fungal cells presents significant challenges, mostly because of the complexity of the fungal cell wall. Targeted therapy requires innovative solutions such as nanoparticle carriers or viral vectors to ensure the modulators reach their intended genomic targets (Zhao et al. 2023; Fujita and Fujii 2022).

**Figure 4:**
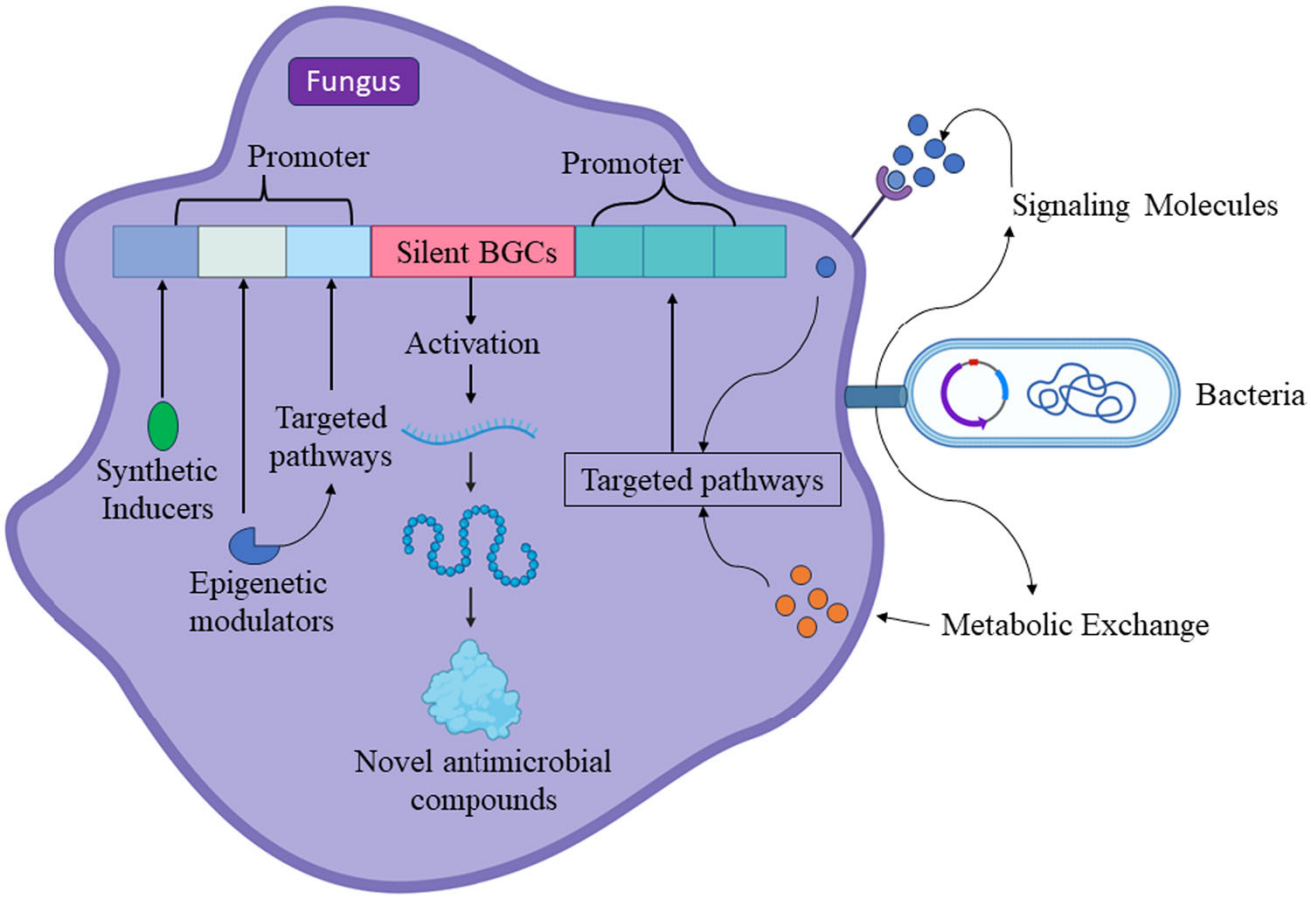
Mechanisms of enhanced antibiotic productions in fungi through synthetic molecules and fungal–bacterial interactions.

Figure 4 highlights how synthetic induces can affect QS and other regulators that may boost antibiotic production in fungi.

### 2.9 Fungal–Bacterial Microbiome Interactions in Antibiotic Discovery

#### 2.9.1 Cross‐Talk Through Signaling Molecules and Coculture Systems

Cross‐talk through signaling molecules plays a crucial role in modulating antibiotic production in the complex microbiome. Volatile organic compounds (VOCs) (Vaishnavi et al. 2021) and diffusible signaling factors (DSFs) (Yetgin 2023) significantly impact fungal–bacterial interactions by regulating gene expression and metabolic pathways. The interaction between the bacterium *Serratia plymuthica* and the fungal pathogen *Fusarium culmorum* demonstrated that fungal VOCs can induce changes in bacterial gene and protein expression related to motility, signal transduction, and secondary metabolites (Schmidt et al. 2017; Almeida et al. 2023). Again, DSFs from bacteria such as Burkholderia species activate fungal non‐ribosomal peptide synthetase genes, boosting antibiotic synthesis through pathways like the target of rapamycin, high osmolarity glycerol, and calcineurin signaling pathways (Bach et al. 2022; Chen et al. 2021; Tran et al. 2020). Despite understanding these interactions, the exact molecular mechanism remains unclear, offering an opportunity for advanced research.

Furthermore, the fungal–bacterial coculture system also significantly influences antibacterial compound variation and production. For instance, coculturing fungal species with carbapenem‐resistance *Klebsiella pneumoniae* resulted in bacterial growth inhibition and the induction of fungal secondary metabolites with antibacterial properties (Moubasher et al. 2022). The study suggested that bacteria under chemical stress showed variable responses and induced fungal secondary metabolites. Another recent study indicated that the Streptomyces‐fungus interaction zone in cocultured growth results in antibacterial activity under certain conditions (Nicault et al. 2021). However, the core mechanism and process of activating genes in these interactions are still a growing field of study. Future research could focus on optimizing coculture conditions and employing advanced geniting and metabolic engineering techniques to further maximize the yield of novel antimicrobial compounds.

#### 2.9.2 Bacterial‐Fungal Metabolic Exchange in Complex Microbiome

The metabolic exchange between fungi and bacteria involves the transfer of metabolites that can dominantly influence cellular activity and expression (Baby and Thomas 2021). Bacteria can provide essential precursors for fungal antibiotic biosynthesis, likewise, bacterial production of short‐chain fatty acids (SCFAs) can serve as a precursor for fungal metabolite production, while fungal metabolites can inhibit bacterial competitors (Luu et al. 2023; Gerke et al. 2021; Sun et al. 2022). Metabolic pathways such as the TCA cycle, glycolysis, and amino acid biosynthesis are often involved in these exchanges (Nielsen et al. 2019). As previously discussed, specific molecules such as farnesol and tyrosol play concentration‐dependent crucial roles in these interactions. A high concentration of farnesol reduces bacterial survival and increases the activity of antimicrobial compounds, whereas moderate levels enhance bacterial tolerance (Kong et al. 2017). Tyrosol inhibits virulence factors in *Pseudomonas aeruginosa* and disrupts biofilm formation in Streptococcus mutants. Additionally, various active compounds have been discovered through fungal–bacterial co‐cultural systems as the result of metabolic exchange (Arias et al. 2016; Abdel‐Rhman et al. 2015). Notable examples include pestalones an effective bioactive compound against drug‐resistance bacteria, and glionitrin A, an antitumor metabolite (Zhang and Straight 2019).

Such findings suggest that exploring bacterial‐fungal interaction can lead to the discovery of new antibiotics. Future research could focus on optimizing these metabolic interactions through advanced techniques like isotope labeling and metabolomics to track metabolic fluxes and elucidate the pathways involved (Takahashi et al. 2022). Developing fungal strains with enhanced resistance to bacterial degradation enzymes or modifying antibiotic molecules to evade degradation can further improve yields.

### 2.10 Underexplored Fungi as a Frontier for Novel Antibiotic Sources

Although much attention has been given to common fungal genera like *Penicillium* and *Aspergillus*, vast areas of fungal biodiversity remain poorly explored, often referred to as “fungal dark matter” (Naranjo‐Ortiz and Gabaldón 2019). This includes rare, extremophilic, or slow‐growing fungi from underrepresented phyla like *Basidiomycota, Zygomycota, or Glomeromycota*, many of which inhabit unique ecological niches such as deep‐sea sediments, arid deserts, high‐altitude soils, or symbiotic environments (Rämä and Quandt 2021). Rare and extremophilic fungi, such as deep‐sea isolates and species from underexplored phyla like *Basidiomycota* or *Glomeromycota*, have recently shown promise in producing unique antibiotic scaffolds (Sista Kameshwar and Qin 2019). For instance, novel antimicrobial metabolites have been isolated from marine‐derived *Emericellopsis* and cold‐adapted fungi from Arctic soils, suggesting these niches may harbor untapped chemical diversity (Pan et al. 2024; Agrawal et al. 2023). However, accessing and cultivating these rare fungi is often challenging due to limitations in growth conditions, unculturability, or lack of genomic references. To overcome this, researchers are increasingly using phylogenetic and phylogenomic approaches to trace conserved BGCs across fungal lineages (Ziemert and Jensen 2012; Pizarro et al. 2020), enabling targeted strain selection. Tools like ITS‐guided fungal barcoding (Raja et al. 2017), metagenomic reconstruction, and single‐cell genomics now allow us to detect and classify these fungi even without traditional cultivation. Likewise, metagenomic methods have led to the discovery of novel antibiotics like malacidin and eradacin (Baby and Thomas 2021). By using metagenomic libraries, particularly fosmid‐ and cosmid‐based, researchers can retrieve large DNA fragments that encode entire biosynthetic pathways, aiding in the discovery of novel enzymes and antimicrobial compounds (Mahapatra et al. 2020). Combining these methods with network‐based BGC clustering, AI‐driven genome mining, and dereplication pipelines can systematically narrow down promising candidates for antibiotic production. As these strategies mature, they offer a clearer path for discovering novel antibiotics from the fungal kingdom's most elusive members.

### 2.11 Technological Advancement and Opportunities for Future Development

**Figure 5:**
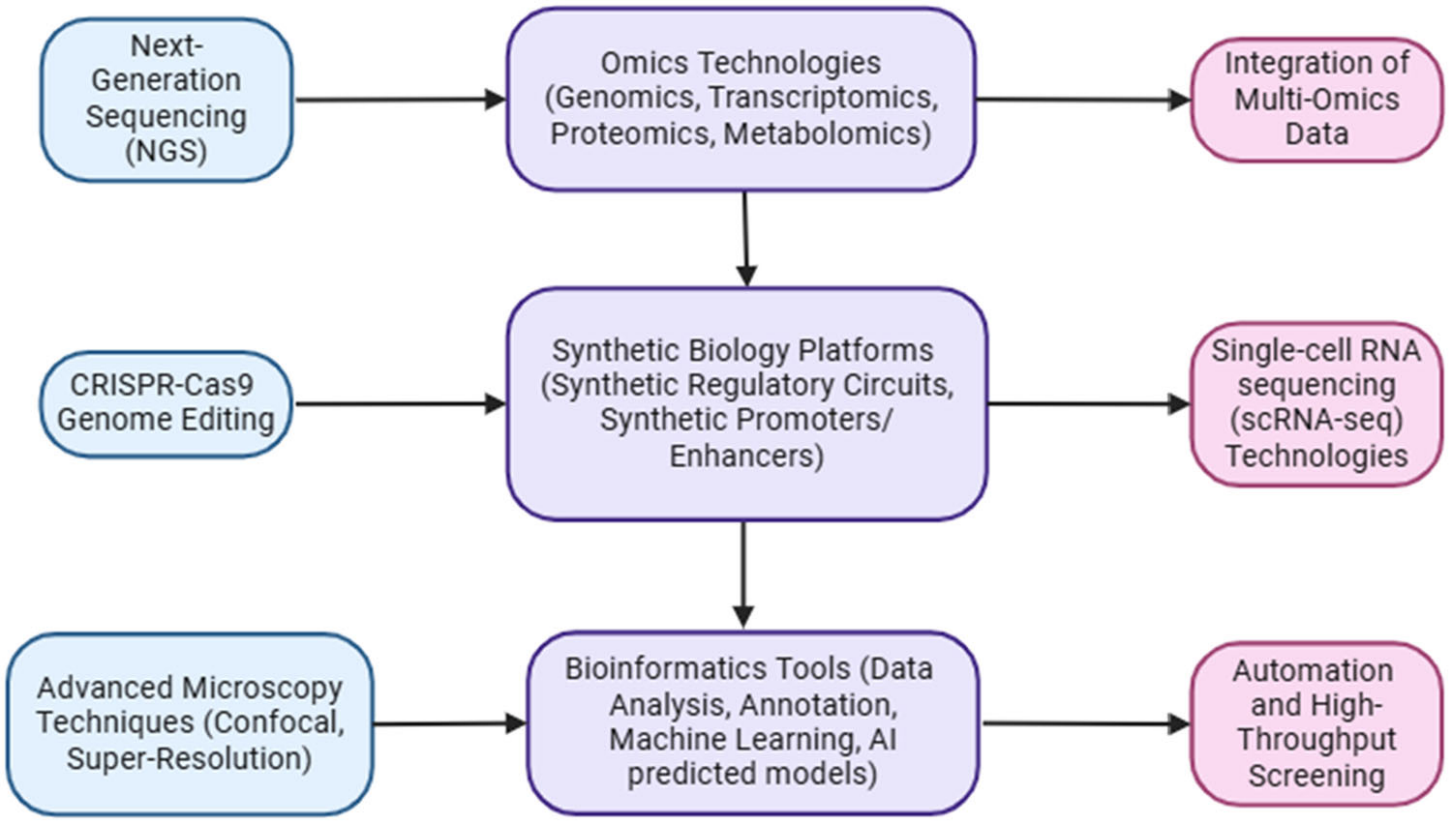
Integration of advanced technologies for exploring fungal microbiome for novel antibiotic discovery.

The field of fungal microbiome or mycobiome research for antibiotic discovery has seen remarkable technological advancements. By leveraging these innovations collectively, researchers can address existing challenges and fully exploit the antibiotic‐producing potential of fungal microbiomes (Ribeiro da Cunha et al. 2019). Next‐generation sequencing allows for comprehensive analysis of fungal genetic materials; concurrently, the integration of multi‐omics (genomics, transcriptomics, proteomics, and metabolomics) provides a holistic understanding of fungal metabolism and secondary metabolite production (Palazzotto and Weber 2018; Crofts et al. 2017). This combination enables the identification of new BGCs and regulatory elements involved in antibiotic production. Building on this, CRISPR‐Cas9 genome editing facilitates the precise manipulation of fungal genomes to enhance NP biosynthesis, while synthetic biology platforms design synthetic regulatory circuits and promoters or optimized gene expression (Tong et al. 2023; Song et al. 2022; Singh et al. 2023). Similarly, single‐cell RNA sequencing (scRNA‐seq) reveals the heterogeneity of fungal populations and their regulatory mechanism (Gasch et al. 2017). Advanced microscopy techniques, coupled with bioinformatic tools, allow detailed visualization and analysis of fungal structures and interactions, aiding automation and high‐throughput screening to further streamline the process of identifying promising fungal strains with antibiotic activity (Scanlon et al. 2014; Carey and Heidari‐Torkabadi 2015). On the contrary, artificial intelligence (AI) is revolutionizing antimicrobial discovery by addressing key challenges such as the lengthy and costly development process, low success rates, and the urgent need for novel antibiotics due to rising antibiotic resistance (Lluka and Stokes 2023; Jiménez‐Luna et al. 2021). Figure 5 presents a flowchart that outlines the technological sources and opportunities for future development in harnessing fungal microbiomes for antibiotic discovery.

AI techniques, particularly machine learning algorithms, are being employed to reinvigorate traditional antibiotic discovery methods like natural production exploration and small molecule screening. For instance, deep neural networks and multi‐layer perceptrons are used for ligand‐based virtual screening to classify compounds as “active” or “inactive” (Wong et al. 2024; Melo et al. 2021; Oladunjoye et al. 2022). AI also facilitates the discovery and optimization of antimicrobial peptides by using sequence‐based features and evolutionary algorithms to generate peptide libraries with promising biological activity (Agüero‐Chapin et al. 2022; Ruiz Puentes et al. 2022). Moreover, AI‐driven models can predict the mechanism of action (MOA) of new compounds with high accuracy, as demonstrated by the CoHEC model, which uses transcriptome responses to identify novel MOAs in antibiotics (Espinoza et al. 2021). Additionally, AI's role in de novo molecular design and the prediction of drug‐likeness traits further streamline the development pipeline, making it more efficient and cost‐effective (Qureshi et al. 2023; Patel and Shah 2022).

The synergy of these technological advancements holds great promise for optimizing conditions for antibiotic production and discovering novel antibiotics essential for combating antibiotic resistance. Open access to high‐quality screening datasets and interdisciplinary collaboration is crucial for maximizing the potential of these technologies by ensuring a sustainable and effective response to antibiotic resistance globally.

## 3 Conclusion

Fungi have long proven their value as a source of antibiotics, yet much of their biosynthetic potential remains untapped. In this review, we highlighted the historical contributions of the fungal microbiome to antibiotic discovery and explored how modern tools are opening new possibilities. Advances in genetic engineering, particularly CRISPR/Cas9 systems, promoter engineering, and synthetic biology approaches, are helping researchers unlock silent BGCs and access new secondary metabolites. At the same time, innovations in cultivation methods, such as in situ separation techniques, are making it more feasible to scale up fungal production for industrial applications. Understanding fungal communication systems, such as QS, and exploring fungal–bacterial interactions are emerging as powerful strategies to boost metabolite production. Moving forward, combining genetic, technological, and ecological approaches, along with stronger interdisciplinary collaboration, will be key to overcoming current limitations. Unlocking the full potential of the fungal microbiome could provide urgently needed solutions to the growing threat of antimicrobial resistance and safeguard public health for generations to come.

## Author Contributions

**Md. Sakhawat Hossain:** conceptualization, data curation, writing – original draft, formal analysis. **Md. Al Amin:** conceptualization, data curation, writing – original draft, formal analysis. **Sirajul Islam:** data curation, validation, formal analysis. **Hasan Imam:** formal analysis, validation, data curation. **Liton Chandra Das:** data curation, formal analysis, validation. **Shahin Mahmud:** conceptualization, supervision, writing – review and editing.

## Ethics Statement

The authors have nothing to report.

## Conflicts of Interest

The authors declare no conflicts of interest.

## Data Availability

The data that support the findings of this study are available on request from the corresponding author. The data are not publicly available due to privacy or ethical restrictions.
